# Highly Pathogenic Avian Influenza Virus (H5N1) in Domestic Poultry and Relationship with Migratory Birds, South Korea 

**DOI:** 10.3201/eid1403.070767

**Published:** 2008-03

**Authors:** Youn-Jeong Lee, Young-Ki Choi, Yong-Joo Kim, Min-Suk Song, Ok-Mi Jeong, Eun-Kyoung Lee, Woo-Jin Jeon, Wooseog Jeong, Seong-Joon Joh, Kang-seuk Choi, Moon Her, Min-Chul Kim, Aeran Kim, Min-Jeong Kim, Eun ho Lee, Tak-Gue Oh, Ho-Jin Moon, Dae-Won Yoo, Jae-Hong Kim, Moon-Hee Sung, Haryoung Poo, Jun-Hun Kwon, Chul-Joong Kim

**Affiliations:** * Ministry of Agriculture and Forestry, Anyang, South Korea; † College of Medicine and Medical Research Institute of Chungbuk National University, Cheongju, South Korea; ‡ College of Veterinary Medicine of Chungnam National University, Daejeon, South Korea; § College of Veterinary Medicine of Seoul National University, Seoul, South Korea; ¶ Bioleaders Corporation, Daejeon, South Korea; # Korea Research Institute of Bioscience and Biotechnology, Daejeon, South Korea; 1 These authors contributed equally to this work.

**Keywords:** Highly pathogenic avian influenza, H5N1 virus, South Korea, poultry, wild birds, dispatch

## Abstract

During the 2006–2007 winter season in South Korea, several outbreaks of highly pathogenic avian influenza virus (H5N1) were confirmed among domestic poultry and in migratory bird habitats. Phylogenetic analysis showed that all isolates were closely related and that all belong to the A/bar-headed goose/Qinghai/5/2005–like lineage rather than the A/chicken/Korea/ES/2003–like lineage.

Highly pathogenic avian influenza (HPAI) virus (H5N1) has been detected repeatedly in domestic poultry and wild birds since 1997, and it poses a substantial threat to human health (*1*,*2*). Since the end of 2003, influenza virus (H5N1) strains have spread in an unprecedented manner in many Asian countries, and the outbreaks have resulted in >170 human deaths in Thailand, Vietnam, Cambodia, and Indonesia. These outbreaks have also caused serious economic losses in the poultry industry (www.oie.int), including South Korea in 2003—the first official report of subtype H5N1 in South Korea’s history (*3*).

Since the outbreak caused by subtype H5N1 from migratory waterfowl on Qinghai Lake (QH) in May 2005 (*4*,*5*), outbreaks of QH-like avian influenza virus (H5N1) have been reported in the People’s Republic of China, Mongolia, Russia, Europe, and Africa, and have been ascribed to the migration of wild birds (*6*,*7*). In contrast to virus found in countries on the western side of Qinghai Lake, the Fujian-like avian influenza virus (H5N1) sublineage has predominated in southern China since late 2005 (*8*), but no outbreaks were reported in Far-Eastern Asian countries such as South Korea and Japan until October 2006. Eventually, in November 2006 and January 2007, outbreaks of HPAI (H5N1) occurred in South Korea and Japan. Here we report the second outbreak of HPAI (H5N1) among poultry in South Korea since November 2006 and its relationship with 2 HPAI virus (H5N1) strains isolated from migratory bird habitats (i.e., in the environment).

## The Study 

All of the virus strains from domestic poultry used in this study were isolated by the Korean National Veterinary Research and Quarantine Service (NVRQS) in embryonated eggs that were inoculated with tissues and swab specimens collected from the oropharynx and cloaca of affected birds. Two subtype H5N1 strains were isolated in embryonated eggs that had been inoculated with fecal specimens obtained from migratory bird habitats by Chungbuk National University and Chungnam National University. Viral genes were sequenced and analyzed as described (*9*). The full-length sequences for each segment were used in phylogenetic analyses. Gene sequences determined in this study have been deposited in GenBank under accession nos. EU233675–EU233746.

**Figure 1:**
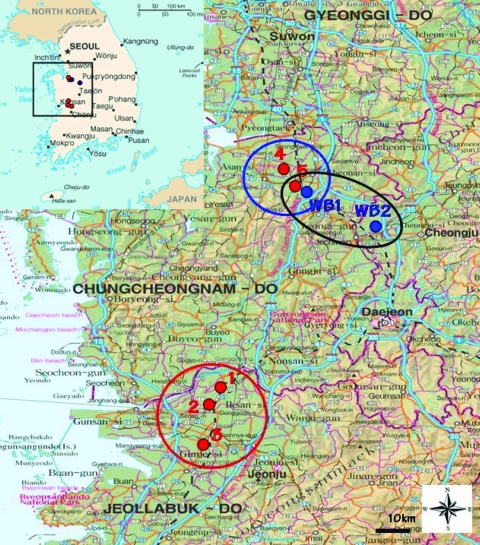
Location of highly pathogenic avian influenza (HPAI) virus (H5N1) outbreaks, South Korea, 2006–2007. Black box on inset shows area of enlargement. Red circle represents outbreaks in Jeollabuk-Do (first, second, and third outbreaks). Blue circle represents those in Chungcheongnam-Do (fourth and fifth outbreaks). Black oval represents regions in which HPAI virus (H5N1) isolates were isolated from migratory bird habitats during this study.

On November 22, 2006, NVRQS confirmed the first case of HPAI (H5N1) at a chicken farm in Iksan, Jeollabuk-Do, in South Korea. The affected flock contained 6,500 chickens and had shown a sudden increase in severe clinical signs and high mortality rate (86%), as reported by farmers and veterinarians (Table). During intensive surveillance in December 2006 within a 10-km radius (the surveillance zone) from the first outbreak farms, we found other HPAI (H5N1)–affected farms at Iksan (chicken farm, 3.4% deaths) and Gimje (quail farm, 4% deaths), Jeollabuk-Do (Figure 1).

**Table:** History of highly pathogenic avian influenza (H5N1) suspected cases, South Korea, 2006–2007

Date reported	Outbreak no.	Source/ breed*	Age, wk	Region	No. animals/farm	Clinical signs	Isolate
2006 Nov 22	1	Chicken/BB	45	Jeollabuk Iksan	13,000	Depression, death	A/chicken/IS/2006 A/chicken/IS2/2006
2006 Nov 27	2	Chicken/BB	40	Jeollabuk Iksan	12,000	Depression, death	A/chicken/IS3/2006
2006 Dec 10	3	Quail	16–28	Jeollabuk Gimje	290,000	Death	A/quail/KJ4/2006
2006 Dec 21	4	Duck/B	30	Chungcheongnam Asan	9,000	Decrease in egg production	A/duck/Asan5/2006 A/duck/Asan6/2006
2007 Jan 20	5	Chicken/L	32	Chungcheongnam Cheonan	30,000	Depression, death	A/chicken/CA7/2007
2006 Dec 21		Environment		Chungcheongnam Cheonan			A/environment/Korea/ W149/2006
2006 Dec 21		Environment		Chungcheongbuk Cheongju			A/environment/Korea/ W150/2006

*BB, broiler breeder; B, breeder; L, layer.

On December 21, 2006, the fourth outbreak of HPAI (H5N1) was confirmed in Asan, Chungcheongnam-Do, in breeder ducks that had shown a severe drop in egg production but no deaths. During intensive observation within the surveillance zone from the fourth outbreak farm, another HPAI (H5N1) outbreak was confirmed in Cheonan, Chungcheongnam-Do, on January 20, 2007, in a layer chicken farm with a 1% mortality rate (Figure 1). Compared with the rates in the first outbreak, the mortality rates in the more recent outbreaks were low (1%–4%) at notification time. This low proportion of deaths could be attributed to the early reporting system between the farmers and NVRQS, when the mortality rate reached ≈1% of the flock on poultry farms, and to the culling of flocks on reverse transcription–PCR confirmation (usually within 1 day) to prevent the spread of the disease. This could have limited the recorded observations when the infecting influenza virus was eliminated before the full extent of its pathogenicity could be manifested, usually after several days of infection.

## Conclusions 

Great interest has been focused on the role of migratory birds in the spread of H5N1 subtype and the exchange of virus strains between domestic and wild birds in Asia. Therefore, we surveyed avian influenza virus in migratory birds in South Korea to investigate whether the HPAI (H5N1) outbreaks in domestic poultry bore any relationship to bird migration in the same region. During our routine survey for influenza activity in migratory bird habitats, on December 21, 2006, 2 distinct subtype H5N1 strains were isolated from fecal samples from 2 migratory bird habitats—one near the first outbreak farm in Chungcheongnam-Do, and the other from a stream in Chungcheongbuk-Do (Figure 1).

**Figure 2:**
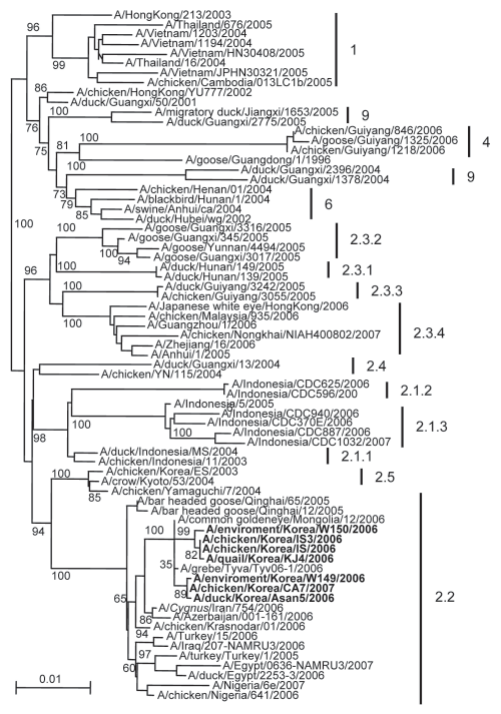
Phylogenetic trees for hemagglutinin (HA) genes of Korean influenza virus (H5N1) isolates from wild birds and poultry farms during 2006–2007. The DNA sequences were compiled and edited by using the Lasergene sequence analysis software package (DNASTAR, Madison, WI, USA). Multiple sequence alignments were made by using ClustalX (*10*). Rooted phylograms were prepared with the neighbor-joining algorithm and then plotted by using NJplot (*11*). Branch lengths are proportional to sequence divergence and can be measured relative to the scale bar shown (0.01-nt changes per site). Branch labels record the stability of the branches >1,000 bootstrap replicates. The tree was produced by referring to the proposed global nomenclature system for influenza virus (H5N1) (www.offlu.net). **Boldface** indicates isolates tested in the current study.

Our phylogenetic analysis of the hemagglutinin (HA) genes of all Korean isolates showed that the isolates belong to the A/bar-headed goose/QH/65/2005 (QH/2005)–like lineage that caused an outbreak among wild birds at Qinghai Lake in China during 2005, rather than the first HPAI (H5N1) lineage (A/chicken/Korea/ES/2003) that infected farms in Korea in 2003 (Figure 2). Notably, the 2 isolates from migratory bird habitats were closely related to the H5N1 subtype poultry virus strains: A/environment/Korea/W149/2006 was similar to the viruses that occurred in Chungcheongnam-Do, and A/environment/Korea/W150/2006 was similar to viruses that affected birds in Jeollabuk-Do. However, all H5N1 subtype virus strains have a series of basic amino acids at the HA cleavage site (PQGERRRKKR/G), which is a characteristic of influenza viruses that are highly pathogenic to chickens (*4*,*5*). The intravenous pathogenicity index score of A/chicken/Korea/IS/2006 was 3.0 in chickens.

Phylogenetic analysis of the other genes showed a similar evolutionary pattern to the HA gene tree. All 2006–2007 isolates had a 20-aa deletion in the stalk region (residues 49–68) of neuraminidase (NA) compared with the NA of A/goose/Guangdong/1/96. Analysis of the raw sequencing traces showed no mutations in NA genes of all isolates associated with resistance to NA inhibitors. All 2006–2007 Korean HPAI (H5N1) isolates had glutamic acid at position 92 of the nonstructural protein 1, a position that is related to the ability of H5N1/97 virus to escape the host antiviral cytokine response (*12*), and lysine at position 627 in the PB2 protein, which is commonly observed in QH viruses (*6*). Lysine 627 in PB2 is conserved in authentic human influenza viruses and is associated with high virulence of influenza virus (H5N1) strains in mice (*13*). Analysis of membrane (M) 2 protein sequences showed that none of the 2006–2007 HPAI (H5N1) Korean strains were resistant to amantadine.

In contrast to the 2003 H5N1 subtype isolates (*3*), all of the 2006–2007 H5N1 subtype isolates were QH/05-like strains. Phylogenetic analysis showed that this sublineage spread wildly through Africa and Europe but not in eastern Asia, until the outbreak reported here. We cannot conclude whether wild migratory birds were the origin of the HPAI virus (H5N1) found in poultry or vice versa in South Korea, because the environmental isolates were obtained after the poultry outbreaks. However, the report of outbreaks of similar HPAI virus (H5N1) strains in Japan on January 13, 2007 (www.oie.int/eng) suggested that migratory birds could be a strong mediator for the spread of HPAI virus (H5N1) in South Korea and Japan, as occurred in 2003, because these 2 countries share similar wild bird migration routes. Therefore, continued monitoring of the domestic and wild bird populations is needed to better understand interspecies transmission and to clarify the importance of avian hosts in the ecology of influenza viruses.
